# Assessment of Human Mycotoxin Exposure in Hungary by Urinary Biomarker Determination and the Uncertainties of the Exposure Calculation: A Case Study

**DOI:** 10.3390/foods11010015

**Published:** 2021-12-22

**Authors:** Judit Szabó-Fodor, Mária Szeitzné-Szabó, Brigitta Bóta, Tamás Schieszl, Cserne Angeli, Lucia Gambacorta, Michele Solfrizzo, András Szabó, Melinda Kovács

**Affiliations:** 1 Eötvös Lóránd Research Network MTA-KE-SZIE Mycotoxins in the Food Chain Research Group, Kaposvár Campus, Hungarian University of Agricultural and Life Science, Guba S. u. 40, 7400 Kaposvar, Hungary; bota.brigitta@uni-mate.hu (B.B.); Kovacs.Melinda@uni-mate.hu (M.K.); 2 Institute of Physiology and Nutrition, Kaposvár Campus, Hungarian University of Agricultural and Life Sciences, Guba S. u. 40, 7400 Kaposvar, Hungary; dr.szabomaria@gmail.com (M.S.-S.); schieszltamas95@gmail.com (T.S.); Angeli.Cserne@phd.uni-mate.hu (C.A.); Szabo.Andras@uni-mate.hu (A.S.); 3 Fumizol Ltd., Kisfaludy u. 6/b, 6725 Szeged, Hungary; 4 Institute of Sciences of Food Production (ISPA), National Research Council (CNR), 70126 Bari, Italy; lucia.gambacorta@ispa.cnr.it (L.G.); michele.solfrizzo@ispa.cnr.it (M.S.)

**Keywords:** human exposure, mycotoxin, urine, biomarker, uncertainty, Hungary

## Abstract

Urinary biomarkers of mycotoxin exposure were evaluated in the case of healthy people (*n* = 41) and coeliac patients (*n* = 19) by using a multi-biomarker LC-MS/MS immunoaffinity based method capable to analyse biomarkers of nine mycotoxins, i.e., fumonisin B1 (FB1), fumonisin B2 (FB2), deoxynivalenol (DON), zearalenone (ZEN), ochratoxin A (OTA), Aflatoxin B1 (AFB1), T-2 toxin, HT-2 toxin and Nivalenol (NIV). Urinary biomarker concentrations were used to calculate the probable daily intake (PDI) of fumonisin B1, deoxynivalenol, zearalenone and ochratoxin A and compared with their tolerable daily intake (TDI). The human urinary excretion rate values reported in the literature and the 24 h excretion rate measured in piglets were used to estimate and compare the PDI values of the four mycotoxins. The highest mean biomarker concentrations were found for DON (2.30 ng/mL for healthy people and 2.68 ng/mL for coeliac patients). Mean OTA concentration was significantly higher (p < 0.001) in healthy people compared to coeliac patients. PDI calculated with piglets excretion data exceeded the TDI values by a much smaller percentage than when they were calculated from human data, especially for FB_1_. The uncertainties arising from the different calculations can be well perceived on the basis of these data.

## 1. Introduction

Mycotoxins are low molecular weight organic contaminants produced by various fungal species as secondary metabolites during growth on foods. Aflatoxins, deoxynivalenol (DON), zearalenone (ZEN), fumonisins (FBs) and ochratoxin A (OTA) are the major mycotoxins in agricultural products and food products which are monitored worldwide. These chemicals are responsible for a variety of negative health effects. It is therefore important to estimate exposure and assess the potential impact on public health.

Exposure can be determined using two different approaches, one combining indirect food consumption and contamination data and the other a direct approach based on biomarkers. In both approaches, exposure is expressed as probable daily intake (PDI). Food consumption data and prevalence data for appropriate foods are generally used to estimate population exposure. However, this method cannot estimate individual intake, it usually does not take into account all sources of contamination, so biomarker-based methods are increasingly used to assess blood or urine concentrations of dietary exposure [1,2]. The determination of the maximum tolerable level of mycotoxins is usually based on an estimate of the tolerable daily intake (TDI), with reference to comprehensive food consumption databases. In Europe, cereal products are the main source of exposure to mycotoxins [3], and the level of mycotoxins in the urine is an indicator of the consumption of mainly cereal products contaminated with mycotoxins. A number of studies were performed worldwide in which risk assessment was performed based on urinary biomarkers [4,5,6,7,8,9].

The multi-biomarker approach was used to validate urinary biomarkers of piglets contaminated with a mixture of DON, AFB1, FB_1_, ZEN and OTA at different concentrations. Urine samples were analysed by a multi-biomarker LC-MS/MS method developed and validated to identify and measure biomarkers of these mycotoxins. Mean percentages of dietary mycotoxins excreted in 24 h post-dose urine were 28.5% for DON, 36.8% for ZEN, 2.6% FB_1_, 2.6% for OTA and 2.5% for AFB_1_. A good correlation (r = 0.71–0.76) was observed between the number of mycotoxins administered and the number of relevant biomarkers excreted in the urine 24 h after dosing [10]. Since then, these excretion rates have been used in many studies.

Human biomarker data are expected to provide more accurate data for estimating human exposure. A UPLC-MS/MS multi-biomarker method was used to detect and measure the presence and level of these biomarkers in urine samples from 52 volunteers living in Southern Italy [4]. For OTA and DON, 94% and 40% of volunteers, respectively, exceeded the tolerable daily intake of these mycotoxins. The estimated human exposure to FB_1_ and ZEN in all volunteers was well below the TDI for these mycotoxins.

In a study [5], the exposure of a German population (*n* = 101) to mycotoxins was estimated using an LC-MS/MS urinary multi-biomarker approach. Twenty-three urinary biomarkers were evaluated. Only DON and DON-GlcA (sum of DON-3-GlcA and DON-15-GlcA) were detectable in quantifiable amounts due to the limited sensitivity of the analytical method. The mean daily intake of 0.52 µg DON/kg body weight was calculated. The results of this study suggest that the German population studied had low daily exposure to mycotoxins, but some parts of the population showed a peak above the widely accepted tolerable daily intake of DON.

The BIOMYCO study [6] was designed to assess mycotoxin exposure in Belgian volunteers using urinary biomarkers. Morning urine was collected from a representative portion of the Belgian population according to a standard study protocol in which 155 children (3–12 years of age) and 239 adults (19–65 years of age) were selected based on a random cluster sampling. These urine samples were analysed for the presence of 33 biomarkers. DON, OTA, CIT and their metabolites were the most often detected. Deoxynivalenol-15-glucuronide (DON-15-GlcA) was the main urinary DON biomarker and was found in all urine samples in a range of ng/mL. DON was detected in 70% and 37% of the samples of children and adults, respectively. Based on the urinary levels, the daily intake of DON and OTA was evaluated whereby16–69% of the volunteers possibly exceeded the tolerable daily intake in case of DON and 1% in case of OTA.

In another work [7], a study involving a total of 300 volunteers of adults and children was conducted. OTA and DON were the most frequently occurring mycotoxins in urine, 51 and 63%, respectively, in adults and 96 and 94%, respectively, in children. The PDI values of mycotoxins were below the TDI values except for DON exposure in adults.

Franco et al. (2019) [8] aimed to assess the exposure of Brazilian residents (*n* = 86) to multiple mycotoxins and characterize the associated risk in two sampling time points. Mycotoxins in food and urine samples were determined by LCMS/MS method. Mean PDI values based on urinary biomarkers were 0.001, 84.914, 0.031, 0.377 and 0.002 μg/kg bw/day for AFB1, DON, OTA, FB1 and ZEN, respectively.

The aim of another study [9] was to investigate the exposure to mycotoxins and their association with food intake and background characteristics in adolescents of a national dietary survey. About 3000 children were included in the survey. Urine and blood samples were collected from 1105 participants for mycotoxin biomarker analysis. Mycotoxins were analysed with multi-biomarker methods in urine and serum. From the 35 analytes in urine, the frequency of positive samples was the following: DON 4.8%, DON-15GlcA 9.1%, dihydro-citrinone (DH-CIT, 0.5%), HT-2-glucuronide (HT-2-3-GlcA, 0.1%) and OTA 0.1%. All probable daily intake estimates were below the TDI, except for 1.6% of the volunteers for DON.

The different studies can only be compared if the excretion rates used in the PDI calculation are taken into account. These are summarised in Table 1.

Maize-derived ingredients are frequently used in food formulation. In addition, some population sub-groups (vegans and coeliac persons) may be more exposed compared to the general population.

In this paper, we report the results on the occurrence of DON, OTA, ZEN and FB_1_ in urine samples of 60 (41 healthy and 19 coeliac patients) volunteers. A probable daily intake was calculated for these mycotoxins and compared to the established tolerable daily intake to uncover potential risks among Hungarian adults and children.

## 2. Materials and Methods

### 2.1. Institutional Review Board Statement

The study was conducted in accordance with the Declaration of Helsinki, and the protocol was approved by the Scientific and Research Ethical Committee of the Medical Research Council (Hungary) (ID 32958-3/2017/EKU) and included informed, written consent of all patients.

### 2.2. Participants and Urine Collection

For the evaluation of human exposure to DON, FB1, ZEN and OTA, 60 individuals (41 healthy and 19 coeliac patients) residing in Somogy, Baranya and Pest counties (Hungary) were invited to participate in the urine sampling. Before starting the experiment, they were invited to answer general questions about their health status. People with signs and/or symptoms of liver or kidney illness or any chronic disease were not included in the study due to potential interferences with the metabolism of mycotoxins and creatinine. 

Each individual was asked to provide a 24 h urine sample. 

All samples were subsequently stored at −20 °C until transportation for biomarker analysis.

Frozen samples were sent, under dry ice, to the Institute of Sciences of Food Production (ISPA) in Bari (Italy) for mycotoxins (ZEN, DON, FB1, fumonisin B2, OTA, T-2 toxin, HT-2 toxin and nivalenol) and metabolites (deepoxy-deoxynivalenol, α-zearalenol, β-zearalenol, aflatoxin M1) determination.

### 2.3. Analysis of Urinary Biomarkers

The following mycotoxins and metabolites were analysed in urine samples: ZEN, DON, deepoxy-deoxynivalenol (DOM-1), FB1, fumonisin B2 (FB2), OTA, α-zearalenol (α-ZOL), β-zearalenol (β-ZOL), aflatoxin M1 (AFM1), T-2 toxin, HT-2 toxin and nivalenol (NIV).

Urine samples were thawed, centrifuged and analysed by using the ultra-performance liquid chromatography–tandem mass spectrometry (LC-MS/MS) method previously described [4,19]. Briefly, 6 mL urine was enzymatically digested with β-glucuronidase/sulfatase type H-2 from Helix pomatia (Sigma Aldrich, Milan, Italy) to hydrolyse glucuronide and sulphate conjugates of mycotoxins and/or their metabolites into free mycotoxins/metabolites. Then the digested urine was diluted with water and purified on a Myco6in1+™ multi-antibody immunoaffinity column (IAC, Vicam, Watertown, MA, USA) connected in tandem with an OASIS^®^ HLB column (Waters, Milford, MA, USA). Therefore, the sample passed through the immunoaffinity column and then through the OASIS column. After complete elution of the sample, the two columns were separated, washed and the analytes were eluted separately from each column. The two purified extracts were gathered together, dried, reconstituted with 200 µL of a mixture of methanol:water:acetic acid (20:80:0.5) and analysed by LC-MS/MS. Matrix matched calibration curves were used for LC-MS/MS analyte quantitation in the purified urine extracts. The analysis was performed on a triple quadrupole API 5000 mass spectrometer (Applied Biosystems, Foster City, CA, USA), equipped with an ESI interfaced with an Acquity UPLC system comprising a binary pump and a microautosampler (Waters). Data acquisition and processing were performed with Analyst version 1.5.1 software (Applied Biosystems). More details, together with chromatographic and mass spectrometric operating conditions, are described elsewhere [4,20].

### 2.4. Creatinine Analysis in Human Urine

Creatinin was determined with a Roche/Hitachi cobas c 501/502 instrument using the Creatinine Jaffé Gen.2 700 test (Roche Diagnostics GmbH, Mannheim, Germany) in the urine samples. 

This kinetic colorimetric assay is based on the Jaffe method. In alkaline solution, creatinine is a yellow–orange complex with picric acid forms. The degree of dye formation is proportional to the creatine concentration of the sample. The test procedure uses a ‘blind measurement’ of it in order to minimise the interference caused by bilirubin reduction.

### 2.5. Statistical Analysis

Statistical analyses were performed using Microsoft Office Excel (2013) and IBM SPSS 20.0 (2012) software. Data processing and the mathematical-statistical calculations were performed using the compare means (Independent Samples *t*-Test, one-way ANOVA with Tukey’s post-hoc test), correlations and descriptive statistics modules.

A value of the limit of detection (LOD)/2 was used for not detected analytes, whereas for values > LOD and <limit of quantification (LOQ), a value of LOQ/2 was used.

The mycotoxin concentrations were normalised with the creatinine level (mycotoxin concentration/creatinine concentration).

### 2.6. Exposure Assessment

The urinary biomarker concentrations measured in this study were used to estimate the probable daily intake (PDI) of each mycotoxin according to the following formula [4].

$$\mathrm{PDI}=C\times \frac{V}{W}\times \frac{100}{E}$$

PDI probable daily intake of mycotoxin (µg/kg body weight);

where:

C human urinary biomarker concentration (µg/L);

V 24 h human urine volume measured for each volunteer (L);

W human body weight measured for each volunteer (kg);

E mean urinary excretion rate of mycotoxin *.

* The calculations were performed in two ways, taking ‘pigs’ and ‘humans’ excretions rates into consideration:

- Data derived after 24 h post dose in piglets ([10]: 36.8% for ZEN, 27.9% for DON, 2.6% for FB1, 2.6% for OTA);
- Excretion rates from human studies: 72.3% for DON [13]; 0,5% for FB1 [15]; 9,4% for ZEN [12]; 2.5% for OTA [18,21].

## 3. Results

In total, 60 urine samples from healthy people and coeliac patients living in Hungary were analysed for the presence of 12 urinary mycotoxins and/or their metabolites. Six out of twelve analytes were detected whereby ZEN, DON, FB1, FB2 and OTA were the most frequently detected. The following mycotoxins and metabolites could not be detected or quantified: DOM-1, AFM1, T-2 toxin and HT-2 toxin. NIV was detected in only one sample.

The concentrations of these mycotoxins are presented in Table 2. The creatinine-normalised toxin concentrations are shown in Table 3.

The maximum biomarker concentrations were found in healthy people, i.e., 26.731 ng/mg of DON followed by FB1 (1.764 ng/mg), OTA (0.582 ng/mg) and ZEN (0.504 ng/mg). From Table 4, it is also evident that urinary concentrations of DON are much higher than those of the other biomarkers.

A significant difference between the two groups of volunteers was experienced only for OTA concentrations (p < 0.001).

In our data, the DON concentration means the sum of free DON plus DON derived from DON-3--glucuronide (DON-3-GlcA) and DON-15-glucuronide (DON-15-GlcA), total ZEN means the sum of urinary concentrations of free ZEN+α-ZOL+β-ZOL plus their glucuronide and sulphate conjugates and OTA means the sum of free OTA plus its glucuronide conjugate.

The estimated daily mean intake (probable daily intake) of the four investigated mycotoxins were calculated and are reported in Table 4 and Table 5. Due to the availability of human excretion rate for the four mycotoxins considered in this study we used the human excretion rate values found in the literature (i.e., 72.3% for DON [13], 0.5% for FB1 [15], 9.4% for ZEN [12] and 2.5% for OTA [18,21]. We compared the PDI data obtained from the human excretion rate to those obtained using the 24 h excretion rate measured in piglets [10].

Based on piglets excretion of FB1 [10], 2.4% of PDI values in healthy people were above its TDI. No coeliac patients were above the TDI for FB1 (Table 4). Much higher percentages of PDI above the TDI of FB1 were found when the human excretion rate of FB1 was used for calculations. In particular, 39.0% of the PDI values in healthy people and 63.0% in coeliac patients were above the TDI of FB1 (Table 5).

According to the excretion rate of piglets, the mean PDI of FB1 was 22.5% of its TDI in healthy people and 21.5% in coeliac patients (Table 4). In contrast, based on the human excretion rate, the mean PDI of FB1 was 117.2% of its TDI in healthy people and 111.6% in coeliac patients (Table 5).

The PDI values of ZEN were above its TDI is 0% of healthy people and for coeliac patients according to piglets and humans excretion rates (Table 4 and Table 5). The mean PDI of DON was 19.5% of its TDI in healthy people and 22.4% in coeliac patients taking into account the piglets excretion rate (Table 4).

When using the human excretion rate, the mean PDI of DON was only 7.5% of its TDI in healthy people (8.7% for coeliac people) (Table 5).

In 1.7% of all subjects studied (*n* = 60), the PDI of FB1 was greater than its TDI by using the excretion rate of piglets and 46.7% by using the human excretion rate, which is a difference of twenty-seven times (Table 4 and Table 5).

As reported in Table 4 and Table 5, similar mean values of PDI of OTA were estimated in healthy people by using piglets and human excretion rates, i.e., 0.144 and 0.150 µg/kg body weight, respectively. The values of PDI of OTA in coeliac patients were also similar by using piglets and human excretion rates, i.e., 0.089 and 0.092 µg/kg body weight, respectively. These values were about 5–9 times higher than the TDI (0.017 µg/kg body weight) established for this mycotoxin, and 100% of volunteers participating in our study exceeded the TDI of OTA regardless of the excretion rate used for PDI calculation. It should be noted that the piglet and human excretion rates used in this study are very similar, i.e., 2.6% and 2.5%, respectively [10,18]. Moreover, the mean PDI values of coeliac patients were lower than those calculated in healthy people, which was statistically significant for OTA (p < 0.0001).

## 4. Discussion

The calculated values of PDI strongly depended on the urinary excretion rate selected for each mycotoxin with the exception of OTA since similar rates were reported for piglets (2.6%) and humans (2.5%) [10,18]. A large number of values above the TDI of FB1 were observed especially when the human excretion rate was used for PDI calculation. The human mean urinary excretion rate of FB1, reported by Riley et al. (2012) [15], is 0.5% of ingested FB1 measured in eight volunteers. Thus, total urinary excretion was less than 1% of the cumulative dose (0.12–0.9%), which was still higher than that reported by van der Westhuizen et al. (2011) [25], i.e., 0.075% (0.054–0.104%). These values are much less than 2.6% reported in 24 h urine of four piglets fed one bolus of contaminated feed [10]. 

Therefore, the percentage of total volunteers (n = 60) with PDI>TDI of FB1 was 1.7% or 46.7% when the considered excretion rate was 2.6% or 0.5%, respectively. In the calculations, we took into account the stricter 1.0 μg/kg bw/day [23] recommendation for the value of TDI. These values would be significantly lower if the WHO/JECFA recommendation (2017) (2 µg/kg bw/day) were taken into account.

As an example, D’Arco et al. (2009) [26] considered the FBs occurrence in products addressed to children or vegans and coeliac persons on the Italian and Spanish markets, reporting a higher incidence of positive samples within the class of organic foods [26]. Among population categories particularly vulnerable to mycotoxins, patients suffering from coeliac disease might be potentially overexposed due to their restricted gluten-free (GF) diet.

Based on human data, the excretion rate of total DON and total ZEN is 72.3% [13] and 9.4% [12], respectively, while these values are 27.9% for total DON and 36.8% for total ZEN according to a piglet study [10].

Gerding et al. (2014) [5] measured mycotoxin exposure in the German population by using a direct method, i.e., measurement of free and conjugated mycotoxins and their metabolites since they did not hydrolyse the urine samples. The most common biomarker of DON was DON-GlcA that was present in 82% of samples, followed by free DON that was present in 29% of the samples. An excretion rate of 68% of total DON was used to calculate the PDI, according to Warth et al. (2013) [12]. This workgroup [12] conducted their study on the kinetics of DON and ZEN in a single healthy 27-year-old male volunteer. The volunteer consumed high-grain meals for 4 days, and urine was collected at a 24-h period. The working group determined a toxin excretion rate of 68% (60–71%) for total DON (15% free DON, 14% DON-3-GlcA and 39% DON-15-GlcA), and this value for ZEN was 9.4% (7–13.2%). Thus, the excretion rate of free DON was determined to be 15%, while the clearance rate of free ZEN was estimated to be 9.4%, but only from data of a single volunteer (n = 1).

In a human study [13] of 35-, 21- and 59-year-old volunteers monitored the first morning urine for 12 days and urine samples were hydrolysed before analysis. The total DON (free DON + glucuronised DON) excretion rate was 72.3% (59.1–85.5%). This is similar to 68% measured by Warth et al. (2013) [12] for total DON but different from the 27.9% reported for piglets [10].

Franco et al. (2019) [8] estimated human exposure in the Brazilian population. The total DON excretion rates considered for women and men were 72% and 50%, respectively (based on [14]); 50% for OTA (based on [27]), 0.5% for FB1 (based on [15]) and 36.8% for ZEN (based on [10]). Thus, results from human and animal experiments were used in the PDI calculation.

Vidal et al. (2018) [14] found a discrepancy between the total DON excretion rate of women and men and free DON was set at 25%, which is much higher than the 15% reported by Warth et al. (2013) [12].

Studer-Rohr et al. (2000) [21] determined the kinetics of OTA involving a single healthy volunteer. About 3% of a single oral dose of tritium-labelled OTA was excreted with the daily urine by a male human during the first 6 days, with a total urinary excretion of 62% of the dose after 75 days.

In the case of free OTA, there is no clearly defined clearance rate in humans. Along with missing data on the temporal variability and possible dose-related effects of renal excretion of OTA in humans, the uncertainty in calculating dietary intake based on biomarker levels is very high for this mycotoxin [18]. 

Attempts were made to estimate daily intake of OTA based on urinary concentrations in spot samples, but the results are considered questionable because of long half-life, strong plasma protein binding and uncertain excretion rates (values from 2.6% to 50% were used) [4,6,7,8,18].

According to Solfrizzo et al. (2014) [4], in Italy, the mean value of estimated PDI (0.139 µg/kg body weight) based on excretion value of piglets is 5.8–127 times higher than the European PDI values estimated with the diet approach. Our data and the calculated values are in great agreement with the data of Solfrizzo et al. (2014) [4]. These data clearly show that the estimated human exposure to OTA is higher when using the biomarker approach compared to the diet approach. According to Solfrizzo et al. (2014) [4], there are two possible explanations for this: (a) the human excretion of OTA is completely different from that of piglets; (b) food diet approach do not cover all sources of OTA exposure.

The largest differences are observed for FB1 and that for ZEN, all the PDI values did not exceed the TDI value of this mycotoxin by using both human and porcine excretion rates. Furthermore, in the case of OTA, all PDI values exceeded the TDI of this mycotoxin in all cases by using both human and porcine excretion rates. The % of samples with PDI > TDI is 100% for OTA and 0% for ZEN.

As an international comparison, our data can be compared with the following studies: 

In the study of Solfrizzo et al., 2014 [4], 94% and 40% of the Italian volunteers exceeded the TDI of OTA and DON, respectively, whereas human exposure to FB1 and ZEN was largely below the TDI of these mycotoxins for all volunteers. In our case, 100% for OTA, 5.0% for DON and 1.7% for FB1 of volunteers exceeded the TDI values using porcine excretion rate. However, it should be noted that Solfrizzo et al. (2014) [4] considered the milder recommendation (2 µg/kg bw/day) regarding the TDI value of FB1, whereas we used a TDI of 1 µg/kg bw/day.

In the study of Gerding et al. (2014) [5], a PDI of 0.52 µg DON/kg body weight was calculated by using a human clearance rate of 68% for DON. In our case, taking into account a human excretion rate of 72.3%, the calculated PDI of DON was lower, i.e., 0.075 µg/kg bw/day for healthy people and 0.087 µg/kg bw/day for coeliac people. 

According to Lemming et al. (2019) [9], in Sweden, based on 1105 urine samples, all PDI estimates were below tolerable daily intakes, except for 1.6% of the participants for DON. According to our data, in the case of Hungarian participants, the PDI of 5% of volunteers exceeds the TDI of DON.

Based on the above, it can be observed that it is worth comparing the various excretion rates published in the literature so far, as even a low percentage difference can result in multiple exposure levels, e.g., for FB1 (0.5% based on [15] or 2.6% based on [10]).

## 5. Conclusions

A multi-mycotoxin exposure was found for all tested volunteers participating in the study. This is the first report on the occurrence of urinary FB1, ZEN, DON and OTA in Hungary.

The estimated PDI values of FB1 largely exceeded the TDI value for this mycotoxin. PDI values are above TDI in 39.0% (63.2% for coeliac patients) in the case of FB1, using the human excretion rate. We found drastic differences in the excretion rates used in the literature and used them to point out how much difference it can cause in the PDI calculation.

With this comparison, we would like to shed light on the direction and need for further research on validated excretion rates and the uncertainties of the exposure calculated in this way.

For plant farmers and the food industry, our practical advice is to use the fungicide concentration and treatment frequency according to the providers’ guidance in practice to prevent mycotoxin-producing mould contamination. It is recommended to keep dry, cool conditions during the storage of cereals.

## Figures and Tables

**Table 1:** Urinary excretion rates of mycotoxins used in human studies that assessed mycotoxin exposure from urinary mycotoxin concentrations.

Human Studies	N. of Volunteers	Excretion Rates used in the Studies	References of Excretion Rate
Solfrizzo et al., 2014 [4]	52	total OTA 2.6%	Gambacorta et al., 2013 [10] (piglets)
		FB_1_ 2.6%	
		total ZEN 36.8%	
		AFB_1_ as AFM_1_ 2.5%	
		total DON 27.9%	
		total DON 50%	Shephard et al., 2013 [11] (humans)
		FB_1_ 0.5%	
Gerding et al., 2014 [5]	101	total DON 68%	Warth et al., 2013 [12] (humans)
Heyndrickx et al., 2015 [6]	394	total DON 72%	Turner et al., 2010 [13] (humans)
Vidal et al., 2018 [14]	30	DON+DON-3-GLC 72%	Turner et al., 2010 [13] (humans)
		OTA 2.6%	Gambacorta et al., 2013 [10] (piglets)
Mitropoulou et al., 2018 [7]	300	total DON 72%	Turner et al., 2010 [13] (humans)
		FB_1_ 0.5%	Riley et al., 2012 [15] (humans)
		ZEN 28.4%	Gambacorta et al., 2013 [10] (piglets)
		total ZEN 36.8%	
Fan et al., 2019 [16]	260	total DON 72% AFB_1_ as AFM_1_ 1.5% OTA 2.5% FB_1_ 1% ZEN 9.4%	Turner et al., 2010 [13] (humans) Zhu et al., 1987 [17] (human) Degen 2016 [18] (human) Riley et al., 2012 [15] (humans) Warth et al., 2013 [12] (humans)
Franco et al., 2019 [8]	86	AFB_1_ as AFM_1_ for women 1.5%	Zhu et al., 1987 [17] (humans)
		AFB_1_ as AFM_1_ for men 1.7%	Zhu et al., 1987 [17] (humans)
		total DON for women 72%	Vidal et al., 2018 [14] (humans)
		total DON for men 50%	Vidal et al., 2018 [14] (humans)
		FB_1_ 0.5%	Riley et al., 2012 [15] (humans)
		ZEN 36.8%	Gambacorta et al., 2013 [10] (piglets)
Lemming et al., 2019 [9]	1105	DON+DON-15-GLC 72%	Turner et al., 2010 [13] (humans)

**Table 2:** Concentration of mycotoxins (ng/mL) in the urine samples of healthy people and coeliac patients.

	*n* Positive	Mean	Median	SD *	Minimum	Maximum
Healthy people						
ZEN	40	0.042	0.029	0.037	0.012	0.174
α-ZOL	34	0.014	0.013	0.012	0.004	0.063
β-ZOL	8	0.006	0.004	0.006	0.013	0.033
Total ZEN	41	0.062	0.048	0.048	0.008	0.199
DON	18	2.302	0.063	3.734	2.010	18.947
FB_1_	41	0.267	0.194	0.246	0.141	1.525
FB_2_	41	0.060	0.032	0.134	0.008	0.875
OTA	41	0.166 ^a^	0.124	0.155	0.071	1.024
NIV	1	0.475	0.475		0.475	0.475
Coeliac patients						
ZEN	19	0.041	0.034	0.030	0.011	0.121
α-ZOL	11	0.013	0.010	0.018	0.004	0.071
β-ZOL	2	0.005	0.004	0.003	0.013	0.013
Total ZEN	19	0.059	0.040	0.044	0.022	0.152
DON	9	2.681	0.063	3.095	3.876	9.484
FB_1_	19	0.229	0.219	0.055	0.146	0.356
FB_2_	19	0.046	0.029	0.046	0.016	0.226
OTA	19	0.089 ^b^	0.078	0.044	0.052	0.243

^a,b^ Numbers with different superscripts indicate significant differences (p ≤ 0.001) between groups.* SD standard deviation. DOM-1, AFM1, T-2 toxin and HT-2 toxin were negative in all the samples (LOD DOM-1: 0.136 ng/mL; LOD AFM1: 0.019 ng/mL; LOD T-2 toxin: 0.250 ng/mL; LOD HT-2 toxin: 6.250 ng/mL; LOQ DOM-1: 0.45 ng/mL; LOQ AFM1: 0.063 ng/mL; LOQ T-2 toxin: 0.830 ng/mL; LOQ HT-2 toxin: 20.8 ng/mL) LOD ZEN: 0.001 ng/mL, LOQ ZEN: 0.003 ng/mL; LOD α-ZOL: 0.002 ng/mL, LOQ α-ZOL: 0.007 ng/mL; LOD β-ZOL: 0.008 ng/mL, LOQ β-ZOL: 0.025 ng/mL, LOD DON: 0.13 ng/mL, LOQ DON: 0.42 ng/mL; LOD FB1: 0.005 ng/mL, LOQ FB1: 0.017 ng/mL, LOD FB2: 0.005 ng/mL, LOQ FB2: 0.016 ng/mL; LOD OTA: 0.0003 ng/mL, LOQ OTA: 0.001 ng/mL; LOD NIV: 0.018 ng/mL, LOQ NIV: 0.06 ng/mL.

**Table 3:** Creatinine normalised toxin concentrations (ng/mg) in urine samples of healthy people and coeliac patients.

		*n* Positive	Mean	Median	SD *	Minimum	Maximum
Healthy people	Total ZEN	41	0.105	0.063	0.115	0.0104	0.504
	DON	18	3.632	0.181	6.495	0.034	26.731
	FB_1_	41	0.413	0.278	0.246	0.069	1.764
	OTA	41	0.218	0.192	0.104	0.083	0.582
Coeliac patients	ZEN	19	0.101	0.064	0.110	0.017	0.371
	DON	9	3.540	0.147	5.653	0.043	23.752
	FB_1_	19	0.341	0.299	0.173	0.129	0.793
	OTA	19	0.128	0.100	0.070	0.039	0.332

* SD standard deviation.

**Table 4:** Mean and maximum concentration of mycotoxins in human urine samples collected in Hungary from healthy people and coeliac patients and estimated PDI values based on the urinary excretion rate for piglets [10].

		Positive	Conc. (µg/L)		Pig Urinary Excretion (%)	PDI (µg/kg bw/day)		TDI* (µg/kg bw/day)	PDI/TDI %		n. above TDI	% above TDI
			Mean	Maximum		Mean	Maximum		Mean	Maximum		
Healthy people *n* = 41	Total ZEN	41	0.062	0.199	36.8	0.004	0.020	0.25	1.6	8.0	0	0
	DON	18	2.302	18.947	27.9	0.195	1.617	1.00	19.5	161.7	2	4.8
	FB_1_	41	0.267	1.525	2.6	0.225	1.062	1.00	22.5	106.2	1	2.4
	OTA	41	0.166	1.024	2.6	0.144	1.055	0.017	847.1	6205.9	41	100
Coeliac patients *n* = 19	Total ZEN	19	0.059	0.152	36.8	0.004	0.015	0.25	1.6	6.0	0	0
	DON	9	2.681	9.484	27.9	0.224	1.307	1.00	22.4	130.7	1	5.3
	FB_1_	19	0.229	0.356	2.6	0.215	0.410	1.00	21.5	41.0	0	0
	OTA	19	0.089	0.243	2.6	0.089	0.374	0.017	523.5	2200.0	19	100

* TDI values for mycotoxins: ZEN 0.250 μg/kg bw/day [22]; FB1 1.0 μg/kg bw/day [23]; DON 1.0 μg/kg bw/day [13]; OTA 0.017 μg//kg bw/day (this value derived from 0.120 μg/kg bw/week) [24].

**Table 5:** Mean and maximum concentration of mycotoxins in human urine samples collected in Hungary from healthy people and coeliac patients and estimated PDI values based on the urinary excretion rate of human studies.

		Positive	Conc. (µg/L)		Human Uinary Excretion (%)	PDI (µg/kg bw/day)		TDI * (µg/ g bw/day)	PDI/TDI %		n above TDI	% above TDI
			Mean	Maximum		Mean	Maximum		Mean	Maximum		
Healthy people *n* = 41	Total ZEN	41	0.062	0.199	9.4	0.016	0.080	0.25	6.4	32.0	0	0
	DON	18	2.302	18.947	72.3	0.075	0.624	1.00	7.5	62.4	0	0
	FB_1_	41	0.267	1.525	0.5	1.172	5.524	1.00	117.2	552.4	16	39.0
	OTA	41	0.166	1.024	2.5	0.150	1.097	0.017	882.3	6452.9	41	100
Coeliac patients *n* = 19	Total ZEN	19	0.059	0.152	9.4	0.016	0.061	0.25	6.4	24.4	0	0
	DON	9	2.681	90484	72.3	0.087	0.505	1.00	8.7	50.5	0	0
	FB_1_	19	0.229	0.356	0.5	1.116	2.133	1.00	111.6	213.3	12	63.2
	OTA	19	0.089	0.243	2.5	0.092	0.389	0.017	541.2	2288.2	19	100

* TDI values: see the note of Table 4.

## Data Availability

The data that support the findings of this study are available from the corresponding author upon reasonable request.
